# A novel non-slip banded balloon catheter for endoscopic sphincteroplasty: an ex vivo and in vivo pilot study

**DOI:** 10.1038/s41598-023-31206-6

**Published:** 2023-03-10

**Authors:** Tadahisa Inoue, Hiromu Kutsumi, Mayu Ibusuki, Masashi Yoneda

**Affiliations:** 1 Department of Gastroenterology, Aichi Medical University, 1-1 Yazakokarimata, Nagakute, Aichi 480-1195 Japan; 2 Center for Clinical Research and Advance Medicine, Shiga University of Medical Science, Seta Tsukinowa-Cho, Otsu, Shiga 520-2192 Japan

**Keywords:** Biliary tract, Biliary tract disease

## Abstract

Endoscopic balloon sphincteroplasty is an established procedure for the extraction of bile duct stones. However, the balloon often slips during the inflation process, and its length is an impediment if the distance between the papilla and scope is limited and/or the stone is located close to the papilla. This animal experimental study aimed to evaluate the feasibility of a novel short non-slip banded balloon measuring 15–20 mm in length for sphincteroplasty. The ex vivo component of this study was conducted using porcine duodenal papilla. Miniature pigs were subjected to endoscopic retrograde cholangiography in the in vivo component. The technical success of sphincteroplasty without any slippage was the primary outcome of the study and was compared between cases managed with the non-slip banded balloon (non-slip balloon group) and conventional balloon (conventional balloon group). The technical success rate of the ex vivo component, i.e., absence of any slippage, was significantly higher in the non-slip balloon group than in the conventional balloon group with the 8-mm (96.0% vs. 16.0%, *P* < 0.001) and 12-mm diameter balloons (96.0% vs. 0%, *P* < 0.001). The technical success rate of endoscopic sphincteroplasty without slippage in the in vivo component was significantly higher in the non-slip balloon group than in the conventional balloon group (100% vs. 40%, *P* = 0.011). No immediate adverse events were observed in either group. The slippage rate was significantly lower with sphincteroplasty using a non-slip balloon, despite the balloon length being considerably shorter than that of conventional balloons, demonstrating its potential utility in difficult cases.

## Introduction

The formation of bile duct stones constitutes an extremely common pathological condition worldwide. Stone extraction performed under endoscopic retrograde cholangiopancreatography (ERCP) guidance is widely accepted as the standard first-line modality^1–3^. Endoscopic sphincterotomy and/or balloon sphincteroplasty are essential for the removal of bile duct stones^4^. Balloon sphincteroplasty, including endoscopic papillary balloon dilation (EPBD) and endoscopic papillary large balloon dilation (EPLBD), are established procedures^5,6^, for which various balloon catheters are currently available. However, the balloon often slips during inflation, making it an onerous and tricky task, especially for novice operators. Slippage often requires re-expansion several times, resulting in unnecessary expansion, and may possibly increase the incidence of adverse events such as pancreatitis and bleeding associated with tearing. Slippage may be a greater problem especially in balloon enteroscopy-assisted ERCP, due to the absence of a forceps elevator.

To overcome this issue, we developed a novel balloon catheter, whose function is to prevent slippage during the inflation process. In addition, this mechanism can yield an unprecedentedly short balloon length, which may reduce the expansion of areas that do not require it and make the procedure more straightforward. Therefore, this study aimed to evaluate the feasibility of this novel balloon catheter for EPBD and EPLBD in animal experiments.

## Methods

### Study design

This pilot experimental study was conducted in two stages. Step 1 entailed an ex vivo comparative study using porcine duodenal papilla with some amount of surrounding tissue. Step 2 entailed an in vivo study using ERCP and miniature pigs. The outcomes of sphincteroplasty with the novel non-slip balloon catheter in the treatment cohort were compared to those of the same procedure performed using a conventional balloon catheter in the control cohort. The study was conducted in compliance with all relevant guidelines. The ARRIVE guidelines have been followed for the study. The Intervention Technical Center Animal Welfare Committee approved this study.

### Armamentarium

**Figure 1:**
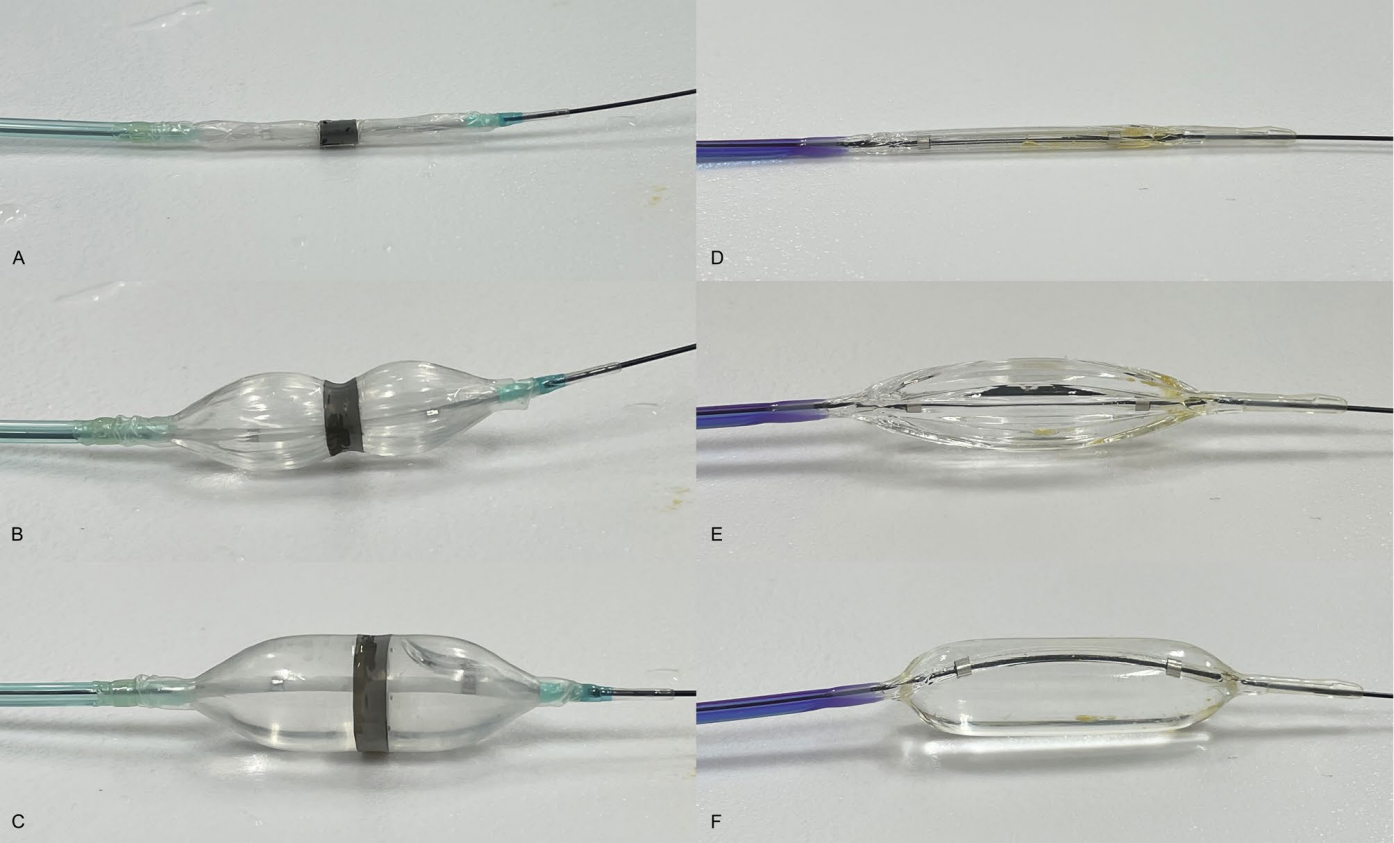
Novel non-slip balloon: before (**A**), during (**B**), and after (**C**) inflation. Conventional balloon: before (**D**), during (**E**), and after (**F**) inflation. A ductile ring-band attached over the non-slip balloon causes the central part of the balloon to expand with delay during balloon inflation, thus preventing balloon slippage.

This study utilized a novel non-slip banded balloon catheter (Japan Lifeline Co., Ltd., Tokyo, Japan), which is equipped with a balloon of 8-mm or 12-mm diameter at the tip. The lengths of the balloon are 15 mm and 20 mm for the 8-mm and 12-mm diameter balloons, respectively. This balloon features a 5-mm ductile ring-band, which is a game changer and is equipped to the center of the balloon; this band ensured that the central part of the balloon would expand with delay during balloon inflation, thus preventing balloon slippage (Fig. 1A–C). The catheter measured 2.35 mm in diameter and 2200 mm in length, with a compatible 0.025/0.035-inch guidewire (Table 1).

**Table 1:** Characteristics of the balloon catheters used in this study.

	Novel non-slip banded balloon		Conventional balloon	
Balloon diameter	8 mm	12 mm	8 mm	12–15 mm
Balloon length	15 mm	20 mm	30 mm	30 mm
Material of balloon	Inner: Nylon Outer: Polyurethane	Inner: Nylon Outer: Polyurethane	Nylon	Nylon
Inflation pressure	8 atm	4 atm	8 atm	3–8 atm
Anti-slip function	Ductile ring-band	Ductile ring-band	None	None
Diameter of catheter	7 Fr	7 Fr	6.4 Fr	7.5 Fr
Length of catheter	2200 mm	2200 mm	1800 mm	1800 mm
Compatible guidewire	0.035 inch	0.035 inch	0.025 inch	0.035 inch
Minimum working channel	3.2 mm	3.2 mm	2.8 mm	3.7 mm

In the control group, a conventional balloon catheter with the same diameter as the novel system (diameter: 8 or 12 mm) and length of 30 mm (CRE RX Biliary Balloon Dilatation Catheter; Boston Scientific, Marlborough, Massachusetts, USA, or REN Biliary Balloon Dilatation Catheter; Kaneka Medix, Osaka, Japan) was used (Fig. 1D–F).

### Experimental procedure

#### Step 1

**Figure 2:**
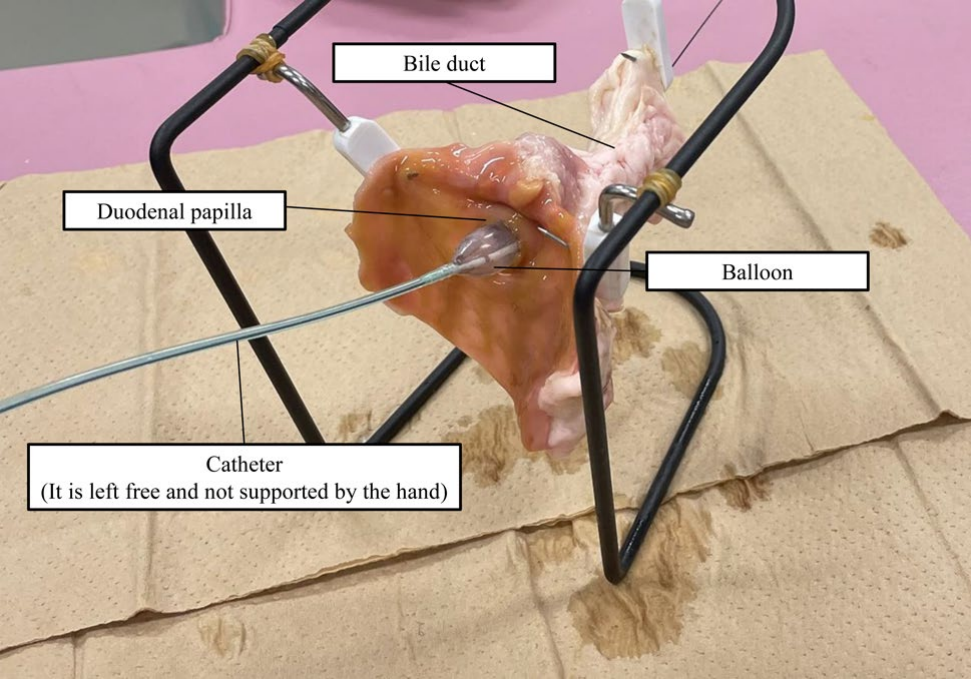
Photographs of the ex vivo experimental setting. Freshly resected porcine duodenum papilla with the bile duct was suspended by a hook. A 0.025-inch standard guidewire was inserted into the bile duct through the papilla, and the balloon catheter was inserted over the guidewire. The center of the balloon was positioned over the papilla, and the catheter was left free (not supported by hand).

Freshly resected porcine duodenum with the bile duct was used for this ex vivo experiment. The duodenum was cut open to expose the duodenal papilla, followed by suspension of the specimen by a hook. A 0.025-inch standard guidewire was inserted into the bile duct through the duodenum papilla, followed by insertion of the balloon catheter over the guidewire. The center of the balloon was positioned over the papilla, and the catheter was left free (not supported by hand) (Fig. 2). Subsequently, the balloon was gradually inflated by the injection of contrast medium in saline with pressure up to 8 atm for the 8-mm diameter balloon and up to 4 atm for the 12-mm diameter balloon. Pressurization was terminated if the balloon slipped. The dilation procedures were conducted 25 times for each of the four balloons (8-mm diameter novel balloon, 12-mm diameter novel balloon, 8-mm diameter conventional balloon, and 12-mm diameter conventional balloon).

A video camera was fixed to the side of the model to record the procedures, which were also evaluated under fluoroscopy. All procedures were performed by a single endoscopist and an unused specimen was used for each procedure, in order to ensure uniformity.

#### Step 2

Miniature pigs weighing 25–45 kg were used for the in vivo experiments. They were premedicated with intramuscular injections of 10 mg/kg ketamine, 2 mg/kg xylazine, and 0.5 mg atropine sulfate. Subsequently, general anesthesia was administered and maintained using 1–3% isoflurane. A TJF-240 duodenoscope (Olympus Medical Systems) was inserted into the duodenal bulb; subsequently, biliary cannulation was performed using a standard catheter with a 0.025- or 0.035-inch standard guidewire. The balloon catheter was inserted over the guidewire, and sphincteroplasty was performed by inflating the balloon up to 8 atm for the 8-mm diameter balloon and 4 atm for the 12-mm diameter balloon. The size of the balloon was selected based on the diameter of the common bile duct measured using cholangiography. After dilation, the presence or absence of adverse events, including bleeding and perforation, was confirmed using endoscopy and cholangiography. All procedures were performed by two endoscopists, and efforts were made to ensure uniformity in each procedure.

### Outcomes

The study outcomes included the technical success rate of sphincteroplasty. Technical success was defined as the successful completion of appropriate papillary dilation solely on the first inflation attempt; full expansion of the balloon was achieved strictly without any slippage, including dislocation of the balloon either into the bile duct or into the duodenum.

The outcomes were compared between the treatment cohort that underwent dilation with the novel non-slip balloon (non-slip balloon group) and the control cohort that underwent dilation with the conventional balloon (conventional balloon group). In the ex vivo experiment, the analysis was conducted separately for the 8-mm and 12-mm diameter balloons. The incidence of immediate adverse events was evaluated during the in vivo experiment component.

### Statistical analysis

The sample size was not calculated since this was a pilot investigation of a novel device. Categorical variables are expressed as numbers and percentages, and differences between them were evaluated using Fisher’s exact test. Continuous variables are expressed as medians and interquartile ranges (IQR). All statistical analyses were conducted using EZR version 1.54 (Saitama Medical Centre, Jichi Medical University, Saitama, Japan)^7^. *P*-values < 0.05 were considered to indicate statistical significance.

## Results

### Step 1. Ex vivo experiment

A total of 100 sphincteroplasty procedures (25 procedures per balloon catheter) were performed using the porcine duodenal papilla models. All applications could be set and conducted without any hindrance. The outcomes of the ex vivo experiments are shown in Table 2.

**Table 2:** Outcomes of the ex vivo experiment.

	Non-slip balloon group	Conventional balloon group	*P* value
8-mm diameter balloon			
Number of successful procedures, n	24	4	
Incidence of slippage, n	1	21	
Median inflation pressure at slippage, atm (IQR)	5 (NA)	1.3 (1.3–1.5)	
Technical success rate, % (95%CI)	96.0 (79.6–99.9)	16.0 (4.5–36.1)	< 0.001
12-mm diameter balloon			
Number of successful procedures, n	24	0	
Incidence of slippage, n	1	25	
Median inflation pressure at slippage, atm (IQR)	2 (NA)	1.1 (1.0–1.2)	
Technical success rate, % (95%CI)	96.0 (79.6–99.9)	0 (0–13.7)	< 0.001

*IQR*, interquartile range; *CI,* confidence interval.

Slippage occurred in 21 cases in the conventional balloon group and 1 case in the non-slip balloon group with the 8 mm-diameter balloon. The median expansion pressure during slippage was 1.3 atm (IQR, 1.3–1.5) in the conventional balloon group, and 5 atm in the non-slip balloon group. The success rates of sphincteroplasty without slippage differed significantly between the conventional and non-slip balloon groups, at 16.0% (4/25) and 96.0% (24/25), respectively (*P* < 0.001).

**Figure 3:**
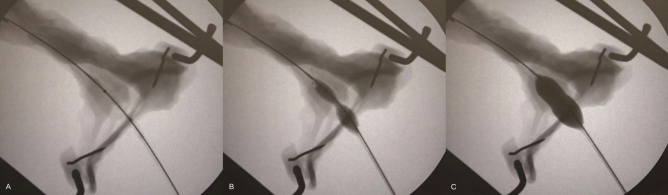
Fluoroscopic images of sphincteroplasty using the novel non-slip balloon in the ex vivo protocol. After the center of the balloon was positioned over the papilla (**A**), the balloon was inflated gradually. The central part of the balloon was expanded in a delayed manner (**B**), and full expansion of the balloon was achieved strictly without slippage (**C**).

Slippage occurred in all cases in the conventional balloon group, and 1 case in the non-slip balloon group for sphincteroplasty with the 12-mm diameter balloon. The median expansion pressure for slippage was 1.1 atm (IQR, 1.0–1.2) in the conventional balloon group, and 2 atm in the non-slip balloon group. The success rate of sphincteroplasty without slippage differed significantly between the conventional and non-slip balloon groups, at 0% (0/25) and 96.0% (24/25), respectively (*P* < 0.001) (Fig. 3 and Video 1–2).

### Step 2. In vivo experiment

**Figure 4:**
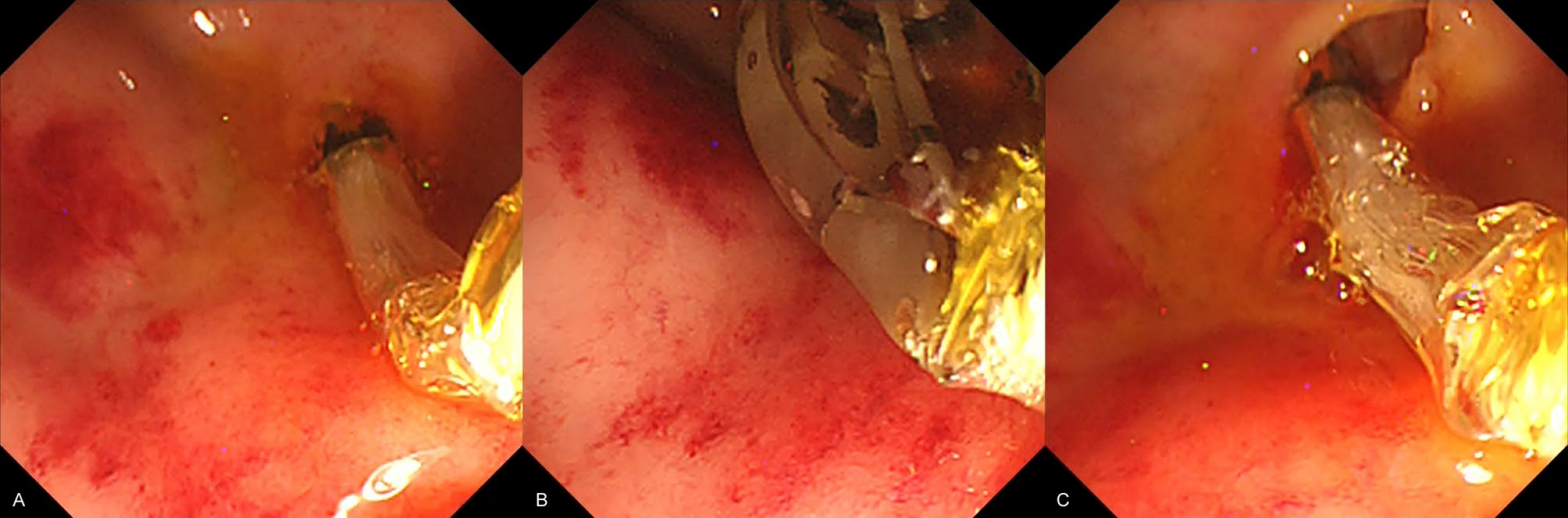
Endoscopic images of sphincteroplasty using the novel non-slip balloon the in vivo protocol. The band part of the balloon was positioned over the papilla (**A**), followed by balloon inflation. Although the lumen of the duodenum was narrow and distance could not be maintained from the papilla, the balloon was short and non-slippery, which enabled full expansion of the balloon without any slippage (**B**) with sufficient papillary dilation (**C**).

ERCP procedures were performed in 20 miniature pigs. The outcomes of the in vivo experiments are presented in Table 3. Biliary cannulation was successful in all cases. The 8-mm and 12-mm diameter balloons were used in 5 cases each in the non-slip balloon group, and 7 and 3 cases in the conventional balloon group, respectively. The technical success rate of sphincteroplasty without slippage in the non-slip balloon group was 100% (10/10); no slippage was observed, and sufficient dilation was obtained in all cases (Fig. 4). The success rate was significantly higher than 40% (4/10) in the conventional balloon group (*P* = 0.011) (Video 3). Endoscopy and cholangiography performed after dilation did not reveal any immediate adverse events, including bleeding and perforation, in either group.

**Table 3:** Outcomes of the in vivo experiment.

	Non-slip balloon group	Conventional balloon group	*P* value
Diameter of the balloon, n			0.650
8 mm	5	7	
12 mm	5	3	
Number of successful procedures, n	10	4	
Incidence of slippage, n	0	6	
Technical success rate, % (95%CI)	100 (69.0–100)	40 (12.2–73.8)	0.011

*CI,* confidence interval.

## Discussion

This study demonstrated that the use of the novel non-slip banded balloon was technically feasible, safe, and accompanied by a very low slippage rate in the ex vivo and in vivo settings. Additionally, the slippage rate with the non-slip banded balloon was significantly lower than with the conventional balloon despite the balloon length being considerably shorter compared to that of the conventional balloon.

Various papillary dilation balloons are currently available; the length of the balloon ranges from 30 to 50 mm. The use of a long balloon can reduce slippage, but it is likely to expand against an unnecessary part in the bile duct wall, possibly increasing the risk of tearing and bleeding. In addition, the procedure becomes difficult in cases where the distance between the papilla and the scope is limited. It may be also difficult to use a long balloon if the stones are located in proximity to the papilla, especially in the event of multiple stone accumulation. Balloons with a short length can be used and adapted in such situations but may risk being encumbered by slippage. Therefore, a shorter balloon with an appropriate anti-slip mechanism may simplify EPBD and EPLBD without any added disadvantage. Prevention of an unwarranted number of dilations or dilation at an unnecessary site may also contribute toward the mitigation of adverse events.

The structure of an elastic and ductile ring-band over the novel balloon enables delayed expansion of the central portion, making it difficult to place the balloon out of position. In conventional balloons, during inflation, the balloon adopts an ellipsoid/ ovaloid geometry, where the largest transverse axis is in the center, which induces slippage towards the edges. Only at the end of filling does the conformation becomes uniform. In this novel balloon, the center of the balloon is the last to be fully inflated, so the balloon always tends to center itself at the point of lowest pressure until the end of inflation, at which point the entire balloon adopts the same conformation as that of the conventional one. In this experiment, slippage did not occur, except for only one case each managed with the 8-mm and 12-mm diameter balloons in the ex vivo experiment; moreover, the results are promising, as the slippage rate was significantly lower than that of the conventional balloon. On the basis of this firm anti-slip mechanism, the length of the balloon can be shortened to 15–20 mm, which can be expanded only at the essential sites. It may also be considered useful in cases where it is difficult to maintain a distance from the papilla. It is often difficult to maintain a distance between the papilla and the scope in pigs, and balloon slippage can occur easily, because of the location of the papilla in the duodenal bulb, the very narrow duodenal lumen, and the highly unstable scope position. However, technical success without slippage was obtained in all cases involving pigs in this study.

The results of this study should be considered in the context of its limitations, which arise from its ex vivo and in vivo design that used pigs for the experimental procedures. The study lacks a blinded validation, raising concerns about potential bias. Although we attempted to simulate the clinical conditions throughout the study, sphincteroplasty in the experimental setting differs from that in the actual clinical setting. In particular, adverse events, including pancreatitis, which is the most important adverse event after sphincteroplasty^8^, could not be evaluated in this setting. Moreover, concomitant use with endoscopic sphincterotomy, which is often performed, was not investigated. Therefore, the actual clinical efficacy and safety of the novel balloon remain uncertain, and the findings of this study must be confirmed by further clinical studies.

Despite these limitations, this is the first study to describe an innovative non-slip banded balloon catheter for endoscopic sphincteroplasty, which yielded promising results. It may be a useful option especially for onerous cases, such as those characterized by a limited distance between the papilla and the scope.

## Supplementary Information

Supplementary Information 1.

Supplementary Video 1.

Supplementary Video 2.

Supplementary Video 3.

## Data Availability

The datasets used and/or analyzed during the current study available from the corresponding author on reasonable request.
