# Supplementary material for: A novel non-slip banded balloon catheter for endoscopic sphincteroplasty: an ex vivo and in vivo pilot study
Source: Sci Rep. 2023 Mar 10;13:4032. doi: 10.1038/s41598-023-31206-6 (PMC10006090; doi:10.1038/s41598-023-31206-6)
Supplement: Supplementary file 1 — Supplementary Information 1. [file 41598_2023_31206_MOESM1_ESM.docx]

**Video 1**

Video of the conventional balloon and novel non-slip balloon in the ex vivo experiments

**Video 2**

Fluoroscopic video of ex vivo experiments using the conventional and novel non-slip balloons

**Video 3**

Endoscopic video of in vivo experiments using the conventional and novel non-slip balloons
